# Reduced functional connectivity between central representations of V1 and foveal-biased face-selective region in central vision loss

**DOI:** 10.1007/s00429-025-02973-x

**Published:** 2025-07-03

**Authors:** Holly D. H. Brown, Richard J. W. Vernon, Heidi A. Baseler, Antony B. Morland

**Affiliations:** 1 School of Psychology, University of Leeds, Leeds, LS2 9JT UK; 2 Department of Psychology, University of York, York, UK; 3 York Neuroimaging Centre, University of York, York, UK; 4 York Biomedical Research Institute, University of York, York, UK; 5 Hull York Medical School, University of York, York, UK

**Keywords:** fMRI, Visual cortex, Central visual deficits, Macular disease, Faces, Places

## Abstract

Individuals with central visual deficits exhibit atrophy of the visual cortex in regions representing the central visual field and show little or no functional response there. Information in the central and peripheral visual field appear to be represented preferentially in extrastriate regions that are selective to faces and places, respectively. We recruited individuals with bilateral macular degeneration (age-related or juvenile) and age-matched sighted controls. We used resting state fMRI (RS-fMRI) to examine functional connectivity between striate (V1) and extrastriate face and place selective areas as it allows better comparison between those with unaffected vision and those with visual loss, whose stimulus related signals are already known to differ from those of controls. Selective deficits emerged in our central loss group, showing reduced functional connectivity between regions with foveal biases (central V1-face area) compared to sighted controls, whereas no such difference emerged in the peripheral biased regions (peripheral V1-place area). This result was evident regardless of whether eyes were closed or open and fixating, but was only significant in the right hemisphere, supporting the functional lateralisation of face processing. This pilot study provides some evidence for reduced functional connectivity between foveal-biased visual areas in central vision loss, suggesting that communication within the posterior visual pathway may be selectively affected in partial vision loss. Functional connectivity differences did not appear to be driven by changes in viewing condition. RS-fMRI is a valuable tool that allows us to explore functional brain changes without the need for retinal input.

## Introduction

Macular Degeneration (MD) is the leading cause of blindness in the developed world, causing a progressive loss of central vision (Office for National Statistics 2018; NICE 2018). Previous work has identified structural and functional changes in the brain associated with MD (Baseler et al. 2011; Brown et al. 2023; Hanson et al. 2019, 2022; Hernowo et al. 2014; Olivo et al. 2015; Prins et al. 2016) but we currently have a limited understanding concerning alterations in functional connectivity, and whether these might be restricted to connections within the cortical representation of damaged visual fields in individuals with MD. Given the established structural changes observed in the posterior visual pathway in MD, it is important to understand how and whether information fed forward to higher-order visual areas when regions earlier in the visual pathway no longer receive input. Cortical regions in the ventral visual pathway that are selective for face and scene processing are known to have a central and peripheral eccentricity bias respectively, and these regions receive input from earlier visual areas (Hasson et al. 2002; Kamps et al. 2020; Levy et al. 2001; Malach et al. 2002; Striem-Amit et al. 2015). One of the most common complaints from those with central vision loss is difficulty identifying familiar faces and interpreting facial expressions (Boucart et al. 2008; Tejeria et al. 2002). This is not surprising given that faces require high acuity to scrutinise - which is progressively lost with MD. There is some evidence suggesting higher-level visual functions such as face and scene processing are affected by sight loss, however, this is largely limited to behavioural studies in partially sighted populations (Peyrin et al. 2017; Roux-Sibilon et al. 2018).

While a number of studies have explored both structural and functional changes across the whole brain in vision loss (Bauer et al. 2017; Bock et al. 2015; Burton et al. 2014; Frezzotti et al. 2014; Sabbah et al. 2016; Striem-Amit et al. 2015), fewer studies have assessed functional connectivity within the posterior visual pathway in cases of partial vision loss (Fleming et al. 2024; Haak et al. 2016; Sabbah et al. 2017; Sanda et al. 2018). Sanda et al. (2018) used resting state functional MRI (RS-fMRI) to explore changes in cortical entropy - a metric associated with synaptic complexity - in both central and peripheral visual field loss. Notably, their findings indicated a possible compensatory increase in cortical entropy - indicative of enhanced connectivity - in regions medial to the mid fusiform sulcus (MFS). The MFS is an anatomical region in the ventral temporal cortex which bisects the fusiform gyrus - a region important for higher level visual processing. Regions lateral and medial to the MFS show a central-eccentricity bias and peripheral-eccentricity bias, respectively (Weiner et al. 2014; Weiner and Zilles 2016). This finding therefore suggests possible compensatory entropy in regions which have more of a peripheral bias - the portion of the visual field largely intact in this central loss patient group (Sanda et al. 2018). How functional connectivity between central and peripheral divisions in V1 and areas involved in higher-order visual processing of face and scenes is affected in MD specifically is largely unknown, and is the focus of the current study.

Determining whether reduced functional connectivity was evident between higher-level visual areas - specifically extrastriate face and place selective areas - with eccentricity biases matching the affected portion in V1 (anatomical representation of the retinal damage) was our main aim for this pilot study. RS-fMRI provides the opportunity to study the organisation of visual cortex and examine functional connectivity between regions of interest (ROI) without the need for visual stimuli or task demands. We predicted that connections between the V1 ROI capturing the central (< 5 degrees) visual field and the corresponding central-biased ventral visual area involved in processing faces (the fusiform face area—FFA) would be reduced in the central loss group compared to sighted controls. We also predicted that the V1 ROI capturing the peripheral (> 5 degrees) and corresponding peripheral-biased ventral visual area and involved in processing places (the parahippocampal place area—PPA) would remain unaffected. Given the evidence of a lateralisation of function for face processing, with the right hemisphere being more dominant (Kanwisher et al. 1997; McCarthy et al. 1997), we examined each hemisphere separately to determine whether functional lateralisation is evident in the functional connectivity data also.

## Methods

### Participants

23 participants were recruited for this study; 16 sighted controls (6 females, mean age = 65.06 years, SD = 10.25, range = 47–82 years old) and 7 individuals with bilateral central vision loss (2 females, mean age = 70.29 years, SD = 11.22, range = 53 to 84 years old) were recruited through advertisements in sight loss support groups in Yorkshire and the York Neuroimaging Centre (YNiC) Participant Pool, University of York. During the screening process, anyone with other eye-affecting or neurological pathologies were excluded from the study. Our central vision loss participants were an opportunity sample and as such, were heterogeneous in their diagnosis, including both age-related and juvenile forms of MD (see Table 1). Given other studies that have compared the different types of MD and found that they show similar results due to the loss of central input, rather than the disease aetiology, we grouped all MD patients together in the current study (Baseler et al. 2011; Brown et al. 2016, 2023; Plank et al. 2011). Due to the COVID-19 outbreak, the study had to terminate early and as such, our central loss group was smaller than our sighted control group (for which data collection was finished in time); we therefore consider this a pilot and feasibility study. While the small number prevents us performing correlational analyses that are likely to be of value in assessing the impact of variable visual field characteristics in our central loss group, our study can still provide valuable insights at the group level. One sighted control was excluded due to poor image quality caused by dental braces, and one central vision loss participant was excluded due to difficulty segmenting structural images (due to image quality) that prevented atlas-based regions of interest from being derived. Fifteen sighted controls and six central loss participants were included in the final analysis. Written informed consent was obtained from all participants. This study followed the tenets of the Declaration of Helsinki with approval granted by the York Neuroimaging Centre (YNiC) Research, Ethics and Governance Committee.

**Table 1:**
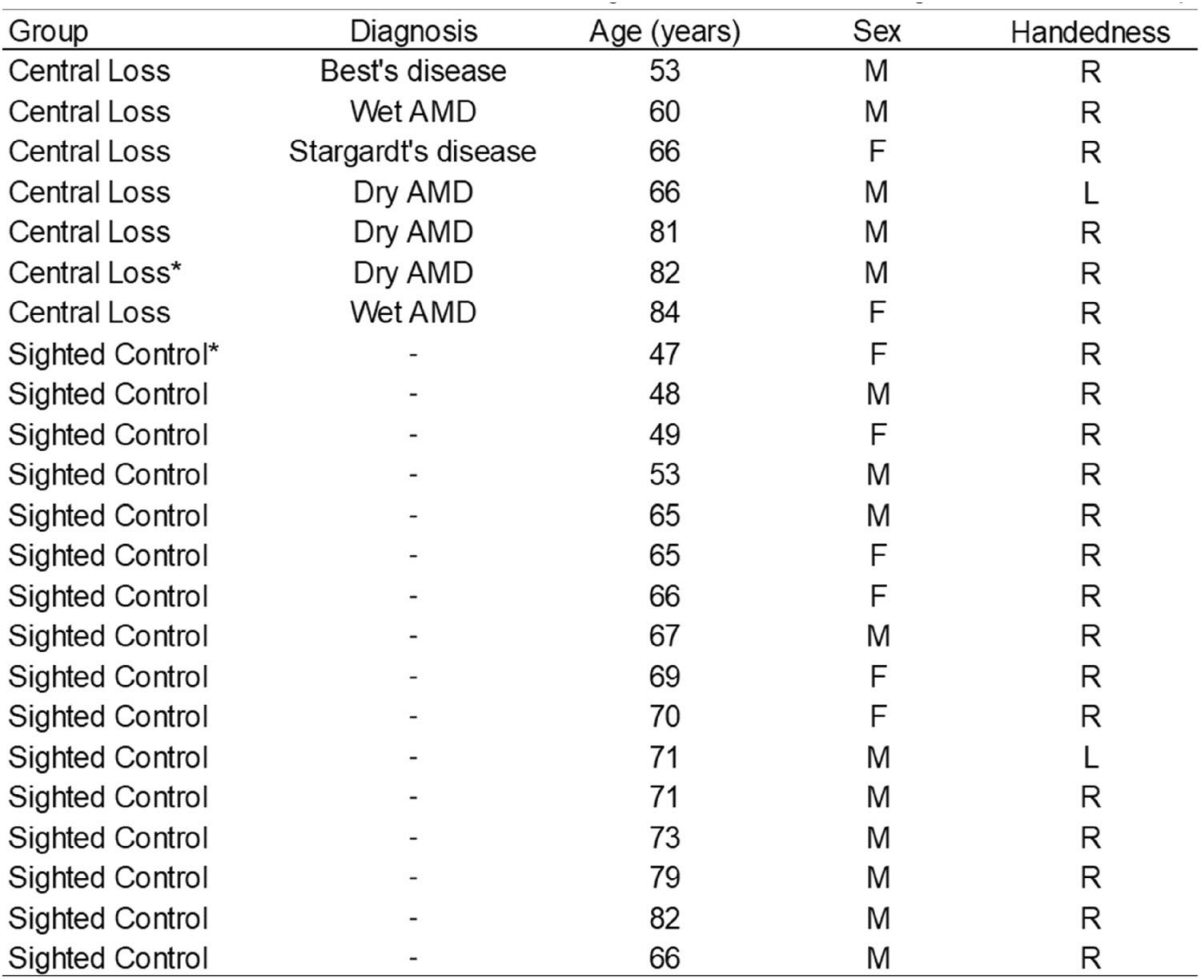
Summary of participant demographics. Diagnosis was reported by the patient at recruitment and was confirmed to be bilateral. AMD = age-related macular degeneration. *Participant excluded

### Design

While we acknowledge that there will be variability in the extent of disease progression in the visual loss group, all members of that group had bilateral central visual loss, which clearly differentiates them from the controls with no visual loss. A between group design is therefore justified. More specifically, we measure functional connectivity between the same regions of the brain - based on atlas definitions - in each participant and compare that connectivity between groups with the appropriate statistical procedures. It is important to note that we specify the regions of interest of the brain based on a straightforward constraint of visual field representation of < 5 deg and > 5 deg in V1. The division secures the essential feature for a group design that each brain is treated in the same way, but variability in the size of the central scotoma in the patient group could reduce the sensitivity of our study. For example, connectivity between the peripheral (> 5 deg) representation in V1 and PPA may be reduced in an individual with a large (> 5 deg) scotoma.

### Scanning procedure

All participants took part in a single scanning session lasting approximately 45 min. The only instructions given to participants were for the two resting state scans (approximately 6 min each), referred to as ‘*Eyes Closed*’ and ‘*Eyes Open*’. The order of RS-fMRI scans was fixed for all participants; given that participants were undertaking a series of short scans, we opted to have all participants complete the eyes closed condition first, when they would be most alert. For the first resting state scan, participants were instructed to close their eyes through the two-way communication system. For those who were hard of hearing, written instructions were presented on the screen in the scanner and participants were asked not to open their eyes again until the sound of the scanner stopped—we confirmed they could indeed hear that the scans had finished. We determined an adequate size and style of font for the instructions prior to going into the scanner, which was particularly important for the visually impaired participants. Participants were also asked to verbally confirm when they had closed their eyes. For the second scan, participants were instructed to keep their eyes open for the duration of the scan and attend to the fixation cross presented on screen somewhere that allowed them to keep their eyes stable, blinking as needed. Participants confirmed verbally that they could see at least a part of the cross. Eye tracking was not used for this study.

### Stimulus presentation

A black fixation cross spanning the full width and height of the screen was presented on a mid-grey screen to participants during the ‘*Eyes Open*’ condition; this waspresented full screen using Microsoft PowerPoint. This is deemed the optimum set up for resting state scans to ensure participants are alert and have something to focus their attention on. Having it full screen ensured both sighted and visually impaired groups could fixate easily, in turn reducing eye movements which could modulate spontaneous activity within the visual network (Yang et al. 2007; Zhang et al. 2015; Zou and Long 2009). The fixation cross was presented to participants using the PROPixx DLP LED Projector (VPixx Technologies, Saint-Bruno, QC Canada; https://vpixx.com/products/propixx/*)*, with 1920 × 1080 Native resolution (HD), 120 Hz refresh rate and a custom in-bore acrylic rear projection screen subtending 40 × 23 degrees of visual angle.

### MRI data acquisition

All structural and functional data were acquired at the University of York Neuroimaging Centre on the 3T Magnetom Prisma MR scanner (Siemens Healthineers, Erlangen, Germany), using the 20-channel head / neck receive-array coil. For T1- and T2-weighted structural scans, we opted for the Human Connectome Project (HCP) recommended protocols (Glasser and Van Essen 2011).

One T1-weighted anatomical image was acquired using a 3D-MPRAGE sequence (TR = 2400ms, TE = 2.28ms, TI = 1010ms, voxel size = 0.8 × 0.8 × 0.8mm^3^, flip angle = 8°, matrix size 320 × 320 × 208, FOV = 256 mm) and one T2-weighted anatomical image was acquired (TR = 3200ms, TE = 563ms, voxel size = 0.8 × 0.8 × 0.8mm^3^, flip angle = 120°, matrix size = 320 × 320 × 208, FOV = 256 mm).

For the functional MRI, we acquired two resting state scans (each approximately 6 min in duration), along with a field map matching the resting state scan parameters (approximately 2 min duration) to help correct distortions caused by inhomogeneity in the magnetic field and improve registration. Two scans using echo planar imaging (EPI) sequence (TR = 1500ms, TE = 31ms, voxel size = 2 × 2 × 2mm^3^, flip angle = 52°, matrix size 120 × 120, 64 slices, FOV = 240 mm, multiband acceleration factor = 4) were acquired.

### Data analysis

#### Structural data

T1 and T2-weighted anatomical scans were processed using the HCP minimal processing stream for structural data (HCP, version 4.0.0). The HCP MRI data pre-processing pipelines use tools from FreeSurfer (Version 6.0) (Van Essen et al. 2012) and FMRIB’s Software Library (FSL; www.fmrib.ox.ac.uk/fsl; Version 5.0) (Jenkinson et al. 2002) to perform cortical reconstruction and volumetric segmentation (Glasser et al. 2013).

### Generating regions of interest (ROIs)

#### Primary visual cortex

**Fig. 1:**
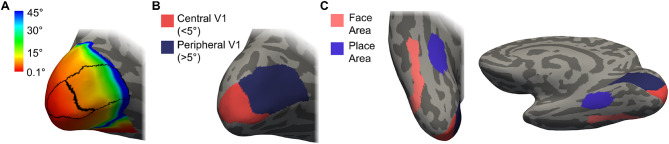
Regions of interest (ROIs) for one example participant, displayed on the FreeSurfer inflated cortical surface. **a**: Two V1 ROIs generated using a retinotopically defined atlas (Benson et al. 2012, 2014), overlaid on the eccentricity map, shown here on the medial surface of the left occipital lobe. **b**: The V1 ROIs in each hemisphere were divided in two—one portion capturing the central visual field (0-5 deg) and one portion capturing the peripheral visual field (> 5 deg). **c**: Our higher-level visual ROIs shown on the ventral surface of the right hemisphere

For each hemisphere, we divided V1 into two parts: a central V1 ROI capturing the visual field representation < 5 degrees of visual angle, and a peripheral V1 ROI capturing beyond 5 degrees (Fig. 1A, B). We used a surface-based atlas approach to ensure we measured from the same brain locations within and across participant groups. An additional advantage is this approach does not require any functional MRI data, only a standard FreeSurfer output directory for each participant for the cortical surface registration. The retinotopic organisation of visual cortex (particularly V1 to V3) is consistent across individuals and when using the cortical surface topology alignment methods (to reduce geometric distortions), there is further evidence to suggest consistency in the size and location of V1 across subjects (Dougherty et al. 2003; Henriksson et al. 2012; Hinds et al. 2008). Benson and colleagues have developed a retinotopic mapping approach that can accurately predict the organisation of visual cortex (primarily V1-V3) simply by registering anatomical data to the cortical surface atlas space (Benson et al. 2012). We applied the Benson atlas (Benson et al. 2014) and restricted V1 based on eccentricity templates provided.

#### Higher-level visual cortex

To find anatomical ROIs capturing face-selective and place-selective regions, we have incorporated both the Human Connectome Project Multi-modal Parcellation version 1.0 (HCP-MMP1.0 (Glasser et al. 2016) and used the version projected onto the FreeSurfer Average Surface Space (https://figshare.com/articles/HCP-MMP1_0_projected_on_fsaverage/3498446*).* Recent attempts using architectural (myelin content, cortical thickness, cortical folding) as well as functional (task-based fMRI, functional connectivity) information to find areal boundaries in the ventral visual pathway have been consistent with previous work focusing on just one property of cortex - typically functional organisation principles. Including a multi-modal approach, the HCP have identified 180 areas per hemisphere. In the ventral visual stream, they have shown regions referred to as the fusiform face complex (FFC) and the posterior inferotemporal (PIT) correspond with the two main clusters of activation in a face-based fMRI study; these aim to capture the functional regions—FFA and OFA—and were combined to create a combined face-selective and central biased representation referred to as the face area. This approach has also been used in other studies (Fleming et al. 2024). For the place-selective and peripheral biased representation, we used a PPA ROI generated by the Grill-Spector group (Weiner et al. 2018) in the FreeSurfer average surface space (http://vpnl.stanford.edu/PlaceSelectivity/*).* Consistent with the functional literature, this ROI was situated on the collateral sulcus (CS), medial to the mid fusiform sulcus (MFS). This ROI also was consistent with the parahippocampal parcels identified in the HCP-MMP1.0 (Weiner et al. 2018). All ROIs were converted from FreeSurfer average surface space to each individual subject’s native anatomical space, and an example viewed on the inflated surface can be seen in Fig. 1C. All ROIs for all participants were visually inspected to ensure successful alignment to the cortical surface. The multi-modal approach to creating this atlas has highlighted a remarkably consistent organisation in the functional representations; in the absence of foveal vision, it enables researchers to still investigate higher-level visual ROIs with eccentricity biases in participants for whom standard functional localiser scans would not be suitable.

#### fMRI preprocessing

Functional MRI data were analysed using FEAT (FMRI Expert Analysis Tool) Version 5.0, part of FSL (FMRIB’s Software Library, www.fmrib.ox.ac.uk/). First, given that functional data acquired with EPI sequences are susceptible to distortions caused by inhomogeneities in the magnetic fields, field maps were prepared by applying FUGUE - an FSL toolbox (https://fsl.fmrib.ox.ac.uk/fsl/fslwiki/FUGUE/Guide#SIEMENS_data*).* This process requires a magnitude image, phase image and the difference of echo times, which was 2.46ms for this protocol. Brain extraction was performed using BET (Smith 2002) on the magnitude image to remove all non-brain voxels (fractional intensity threshold = 0.4). It is important to get a conservative extraction, and so we eroded the image (using default FSL kernel) as an extra precaution.

Preprocessing in FEAT FSL (Worsley 2001) included standard procedures: motion correction using MCFLIRT (Jenkinson et al. 2002), brain extraction using BET (Smith 2002), grand-mean intensity normalisation, B0 unwarping (using field maps described above) and spatial smoothing (Gaussian kernel, 4 mm (double voxel size) FWHM). The data were high pass filtered (gaussian-weighted least squares straight line fitting with sigma = 50.0s) and registration to high resolution anatomical space was carried out using FLIRT (Jenkinson et al. 2002; Jenkinson and Smith 2001). Nuisance regressors were included to help clean up the signal. This included six motion parameter estimates and noise from the white matter and ventricles; to do this, we calculated the mean time series from the white matter and ventricles, creating masks converted to functional space from the FreeSurfer parcellation. These were then regressed out of the functional data using MATLAB (https://www.mathworks.com/products/matlab.html*).* Given our ROIs were generated on the surface, we projected the functional data volume onto the cortical surface as well, using *bbregister* to generate the appropriate registration file (Greve and Fischl 2009).

#### Statistical analysis

For each participant, the mean time series was extracted for each ROI (central V1, peripheral V1, face area and place area), hemisphere and for each condition (eyes open and eyes closed). Eight within-eccentricity correlations were calculated for each participant in total: Central V1 - Face and Peripheral V1 - Place for each viewing condition (eyes open and eyes closed), resulting in four correlations for each hemisphere. All correlations were transformed into Fisher’s Z scores before running statistical analyses. We proceeded with a 2 × 2 × 2 × 2 mixed ANOVA to investigate effects of hemisphere (left versus right), condition (eyes open versus eyes closed), ROI pair (central V1—face versus peripheral V1—place) and group (sighted controls versus central loss). Our reasoning was to seek a four-way interaction as evidence to do four further 2 × 2 ANOVAs to explore group differences for each of our ROI pairs by hemisphere and condition separately. That way, we could determine if there were effects of ROI pair, or group and whether these features were expressed in different hemispheres and under different viewing conditions. As mentioned previously, a functional lateralisation for face processing is also evident in the literature, whereby a bias for face processing is consistently shown in the right hemisphere (Kanwisher et al. 1997; McCarthy et al. 1997), warranting the separate analysis for each hemisphere. We indeed observed this four-way interaction between all of our factors listed above (*F*(1,19) = 4.946, *p* =.038, n_p_^2^ = 0.207) and so proceeded with four further 2 × 2 mixed ANOVAs, with full details shown in Table 2.

**Table 2:**
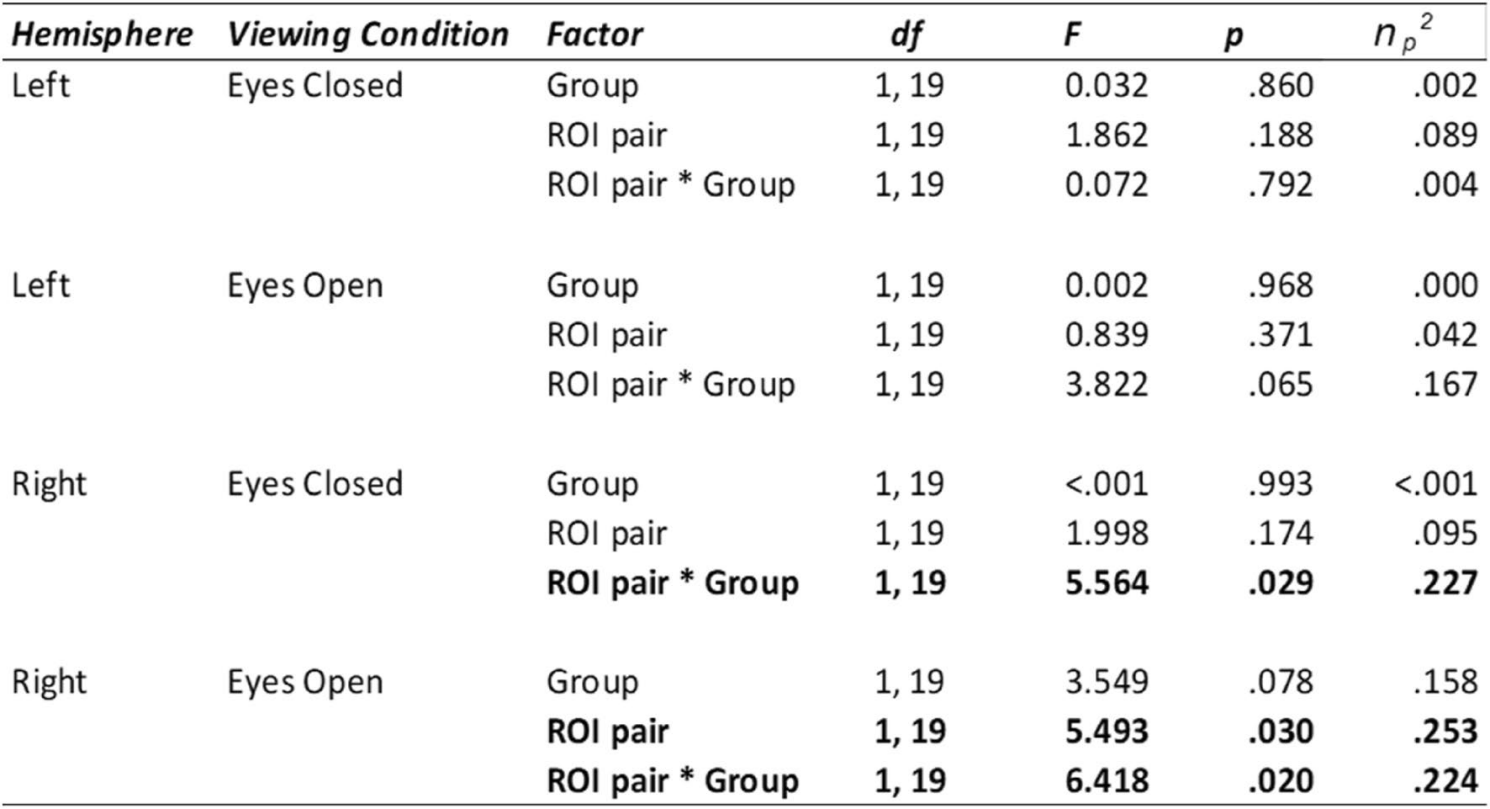
Output from the four 2 × 2 ANOVAs for the within eccentricity correlations. Significant results (*p* <.050) indicated in bold

## Results

**Fig. 2:**
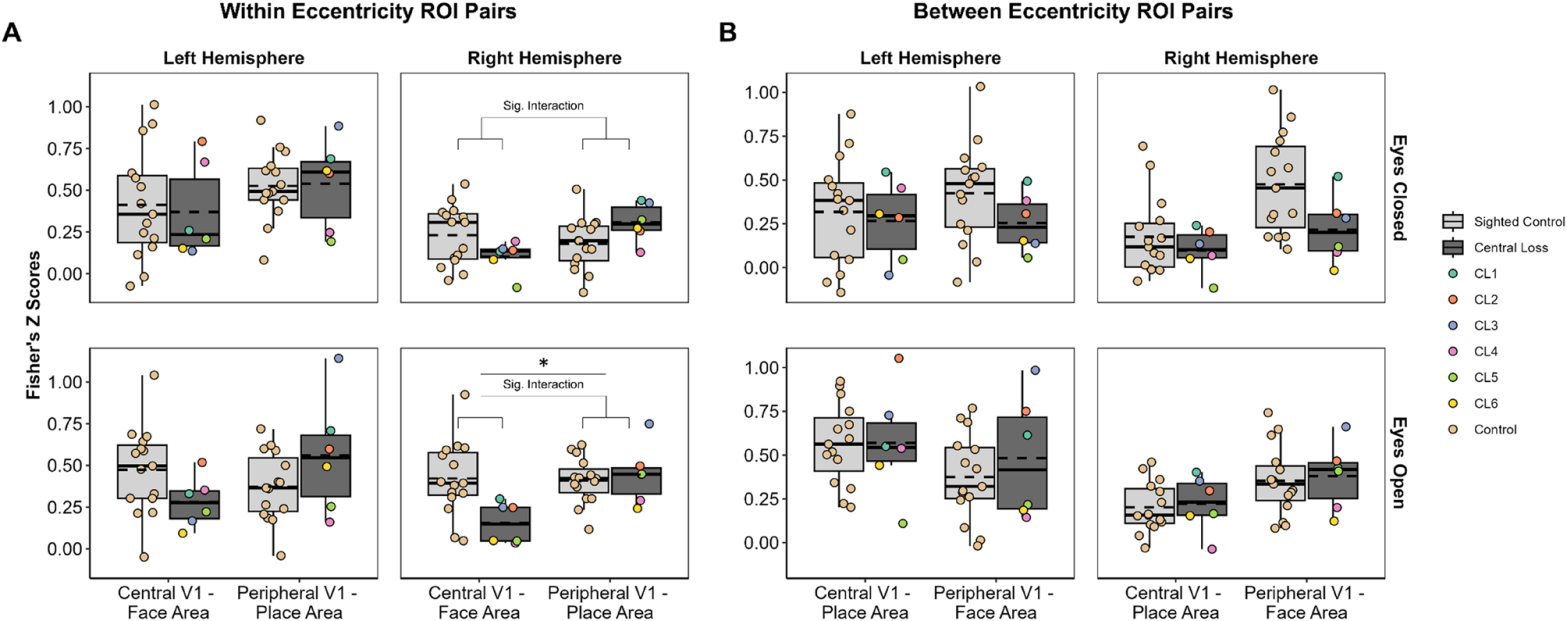
Correlations transformed into Fisher’s Z scores. **a**: Results for within-eccentricity ROI pairs. **b**: Results for between-eccentricity ROI pairs. Box plots: Light grey = sighted control group, dark grey = central loss group. Solid line = group median, dashed line = group mean. Each dot represents a single participant, with central loss patients coloured to allow each individual to be identified across conditions and ROI pairings. **Top row**: Results for *Eyes Closed* condition. **Bottom row**: Results for *Eyes Open* condition

One of our hypotheses was that we would see reductions in functional connectivity for eccentricity-biased correlations in the central loss group. For central loss patients, we expected to see the central V1—face correlations drop. While we acknowledge the lateralised bias of face processing, seen as generally larger in the right hemisphere, there were no expectations concerning hemisphere bias for place processing. Mean Fisher’s Z scores as well as individual data points are shown in Fig. 2A for both hemispheres.

In the left hemisphere, it appears that on average, sighted controls exhibit higher Z scores compared to the central loss group for the central V1—face correlations, whereas the opposite seems to be apparent for the peripheral V1 - place correlations, whereby the central loss group exhibit higher Z scores on average. For the right hemisphere, a similar pattern emerges with sighted controls showing higher average Z scores compared to central loss patients for the central V1 - face correlations. In the peripheral V1—place correlations, we see higher Z scores for the central loss group compared to sighted controls. The group differences appear to be greater in the right hemisphere than the left.

In terms of viewing conditions, results appear consistent across the two conditions, but arguably group differences appear more pronounced in the eyes open condition. Inferential statistical analyses were used to determine if any group differences in the descriptive statistics reported above are significant.

In the left hemisphere for the eyes closed condition, there were no significant main effects for ***group*** (*F*(1,19) = 0.032, *p* =.860, n_p_^2^ = 0.002), ***ROI pair*** (*F*(1,19) = 1.862, *p* =.188, n_p_^2^ = 0.089), nor was there a significant ***ROI pair x group*** interaction (*F*(1,19) = 0.072, *p* =.792, n_p_^2^ = 0.004). For the eyes open condition in the left hemisphere, there were no significant main effects for ***group*** (*F*(1,19) = 0.002, *p* =.968, n_p_^2^ <0.001), ***ROI pair*** (*F*(1,19) = 0.839, *p* =.371, n_p_^2^ =0.042), nor were there any significant ***ROI pair x group*** interactions (*F*(1,19) = 3.822, *p* =.065, n_p_^2^ =0.167). Please see Table 2 for full details.

For the right hemisphere in the eyes closed condition, there were no significant main effects for ***group*** (*F*(1,19) < 0.001, *p* =.993, n_p_^2^ <0.001) or ***ROI pair*** (*F*(1,19) = 0.839, *p* =.371, n_p_^2^ =0.095). However, we did observe a significant ***ROI pair*** x ***group*** interaction (*F*(1,19) = 5.564, *p* =.029, n_p_^2^ =0.227). For the eyes open condition in the right hemisphere, there was no significant main effect of ***group*** (*F*(1,19) = 3.549, *p* =.078, n_p_^2^ =0.158). We did observe a significant main effect of ***ROI pair*** (*F*(1,19) = 5.493, *p* =.030, n_p_^2^ =0.253) and a significant ***ROI pair*** x ***group*** interaction (*F*(1,19) = 6.418, *p* =.020, n_p_^2^ =0.224).

While the within-eccentricity correlations were the main interest for the current study, to increase our confidence in the selective nature of the reduced functional connectivity observed in the right hemisphere, the between-eccentricity correlations were investigated also. We replicated the analysis process described previously for the within eccentricity correlations, and proceeded with a 2 × 2 × 2 × 2 mixed ANOVA to investigate effects of hemisphere, condition, ROI pair (this time using central v1 - place area and peripheral V1 - face area) and group. We did not observe a four-way interaction between all of our factors listed above (*F*(1,19) = 0.064, *p* =.802, n_p_^2^ = 0.003), therefore we did not proceed with the follow-up analyses. In Fig. 2B, the main correlation of interest is the central V1 - place area. Quantitatively, there is little evidence of reduced functional connectivity in the central loss patients compared to the sighted control group for this ROI pair in the right hemisphere in particular.

To summarise, the significant interactions between ***group*** and ***ROI pair*** observed in the right hemisphere for both the eyes closed and eyes open conditions indicate that the group differences depended on the ROI pairing and was driven by lower Z scores in the central loss patients compared to the sighted group in the central V1—face correlation. Further, central loss and sighted groups are similar for the peripheral V1—place correlation, with central loss patients showing a slightly higher mean Z score. This applied to both the eyes open and eyes closed conditions with a greater difference in the peripheral V1—place correlation in the eyes closed condition. While none of our main effects or interactions emerged significant in the left hemisphere, we can see similar patterns to the right hemisphere, largely in the eyes open condition.

## Discussion

The primary aim of this study was to explore how selective visual field deficits impact on functional connectivity within the visual cortex, by examining specific connections between areas with matching eccentricity biases. We predicted that those with central vision loss, resulting from macular degeneration, would show a deficit in functional connectivity between the central visual field representation in primary visual cortex (V1) and the atlas-based ROI capturing the foveal biased and face-selective region in the ventral visual pathway. We also predicted the corresponding peripheral V1 and place-selective, peripheral biased region medial to the mid fusiform sulcus (MFS) would remain unaffected, since this represents the portion of vision largely intact in this patient group. In our central vision loss group, we observed selective deficits in functional connectivity, with reduced functional connectivity evident between central V1 and our face area in the right hemisphere. Despite the small sample size, our pilot study adds value to the current literature as a proof of concept, outlining methods and setting up a priori hypotheses that can be applied to a large sample, while also providing interesting findings.

The interaction observed between group and ROI pair for both viewing conditions was interesting; the central loss group showed a deficit in the central V1—face area correlation as predicted, but for correlations between the peripheral V1—place area, central loss patients were similar to the controls, or, if anything, showed quantitatively larger correlations. First, the interactions only emerged in the right hemisphere, supporting the lateralisation of face processing (Kanwisher et al. 1997). We acknowledge that having one left-handed participant in each group may have diluted the observed effects, however this is in line with the expected number of left-handers observed in the population. Second, no significant main effect of group emerged in our analyses, suggesting that our results cannot be explained by a lack of feedforward input from the central V1 ROI, and central loss patients are not simply showing reduced functional connectivity overall. This is further supported by the between eccentricity data (central V1 - place area in particular) which quantitatively, did not show the same pattern in the right hemisphere. Our findings appear to suggest that this prewired within-eccentricity bias identified may be selectively impacted in this central vision loss group (Kamps et al. 2020; Mahon et al. 2009; Mattioni et al. 2020; Murty et al. 2020; Striem-Amit et al. 2015; van den Hurk et al. 2017). Notwithstanding, one potential limitation of our interpretation concerning the lateralisation of our results being driven by lateralised face networks, is the fact that MD patients frequently adopt a preferred retinal locus (PRL). It is possible that PRLs of our participants could be lateralized to the hemifield contralateral to the hemisphere in which we find differences in connectivity. Unfortunately, we had no data concerning the PRL in this small opportunity sample, so cannot explore this directly.

The peripheral V1- place area functional connectivity remaining comparable between patients and controls supported our second prediction and to an extent, what we see in the literature. Sanda et al. (2018) found greater cortical entropy was reported in region FG1—situated medial to the MFS—in central loss patients. This appears to overlap with peripheral biased representations described previously and is likely situated on or near the PPA ROI used in our study. Interestingly, Sanda and colleagues seem to highlight possible compensation in the peripheral representations in the absence of central vision, whereas we did not see this specifically in our peripheral V1—PPA functional connectivity.

Since completing our study another similar investigation into connectivity has been conducted (Fleming et al. 2024). The study predicted an upregulation in connectivity between early visual areas that represent intact, peripheral representations and FFA, but this was not significant, and our results are consistent with this. There was also no significant decrease in connectivity between central representations in early visual cortex and FFA, a feature that was present in our data. The discrepancy could result from a number of differences in the study designs (for example ROI definitions), but also the disease status and duration of visual loss in the patients.

While Fleming et al., (2024) did not show the predicted upregulation of connectivity from early visual cortex to the FFA, it did detect upregulation in the connectivity between early visual cortex and MT, consistent with the compensatory mechanism that increases the connectivity between the representation of spared visual field in early visual cortex and higher extrastriate regions. A similar finding has also been reported by Sabbah et al. (2017) (see also similar work by Sanda et al. 2018) who showed that in Stargardt’s patients the representation of peripheral visual field locations in early visual cortex have increased connectivity with lateral occipital cortex (a region involved in shape and object perception - (Hasson et al. 2002; Malach et al. 2002; Silson et al. 2013; Vernon et al. 2016). It remains to be seen whether the upregulation of connectivity is limited to LOC and MT, which lie relatively close together, or a more general feature that, because of limited statistical power, has not emerged as significant for other connections (e.g. for the FFA as discussed above). Although our small sample study with a priori hypotheses did not permit exploration of other brain regions, further work on larger cohorts should shed light on this issue. Moreover, future studies could look at participants with peripheral visual loss to understand how connectivity might change in these populations. Our initial intent was to do this, but again recruitment limitations prevented us from doing so.

For this study, we were also limited to recruiting participants through charities and sight loss support groups as opposed to through eye clinics in the NHS, in turn limiting access to clinical measures. Volunteers were asked about their diagnosis upon expressing interest in the study and while thisis not ideal, the important issue is that every patient can be differentiated from the controls on the basis of bilateral central visual loss. This was sufficient for this research question given the shortened recruitment window, but also because we were not implementing interventions or making recommendations for better patient outcomes here. Going forward however, it would be preferable to include assessments of visual function to objectively quantify the extent of vision loss in patients (as in Fleming et al. 2024) and refine our V1 ROI definition The size of central visual field loss likely varied across patients, and so we cannot rule out the possibility of vision loss in our central loss group extending beyond 5 degrees in some individuals. This could have the consequence of masking the upregulation of peripheral connectivity that others have reported (Fleming et al. 2024; Sabbah et al. 2017; Sanda et al. 2018). However, using a set ROI ensures confidence that controls have the same ROI constraints as patients, and supports our logic of having our ROIs capturing the common area of central deficit across patients. Having said this, others have also opted for selecting ROIs with restricted eccentricities as opposed to mapping them explicitly in each participant (Sabbah et al. 2017).

Determining how much RS-fMRI is affected by participant viewing conditions in both participant groups was our second aim. This is a source of ongoing debate in the literature and warrants further investigation. In some cases, literature suggests that asking participants to keep their eyes open is generally optimal as it more accurately reflects our day-to-day viewing experience, but this is in sighted populations (Patriat et al. 2013). Given potential fixation instability in partially sighted groups, for our ‘Eyes Open’ condition, we presented a large fixation cross occupying the full width and height of the screen to give participants a greater chance of finding something to fixate on and without requiring the use of a PRL, as some patients may not have one. We predicted therefore that the biggest difference would emerge in our central loss group, since unstable viewing can lead to spontaneous activation, particularly in the visual network (Haak et al. 2016; Koba et al. 2021; Patriat et al. 2013; Zhang et al. 2015; Zou and Long 2009), but also, visual input will inevitably vary across individuals particularly if they have varying amounts of visual loss. Our results were very similar across viewing conditions however, so in this particular cohort, it did not have a large effect. Despite little change, in order to guarantee spontaneous fluctuations are avoided and to ensure differences in visual input are not driving any responses, it seems opting to have the participant’s eyes closed is the best choice. Other studies exploring RS-fMRI in partially or completely blind individuals (with a sighted control group) do often report having eyes closed and sometimes blindfold participants to reduce any light perception (Aguirre et al. 2017; Dai et al. 2013; Sanda et al. 2018).

The advent of RS-fMRI has allowed for greater exploration of visual cortex in individuals with partial or complete vision loss and can provide further insights into any remaining communication/interactions between areas deprived of retinal input and regions higher up the visual hierarchy (Nir et al. 2006). Assessing both the structure and function of visual cortex in various forms of vision loss is important to understand how and when changes occur, which in turn will aid the efforts of visual restoration. It also provides another tool to help understand the relationship between structure and function in the posterior visual pathway. Whilst it is understood that the underlying architecture and retinotopic organisation in visual cortex remains intact even in cases of congenital vision loss, it does not mean that if vision were to be restored, that the posterior visual pathway would be capable of processing restored retinal input in an appropriate manner, allowing for perception resembling ‘normal’ vision. Patients present us with an interesting case, providing a model of how the visual system works, and how functional connectivity may be altered when there is a change to visual input as a result of eye disease.

## Data Availability

.Participants consented to the use of their data by the investigators and to third parties, but only after the use of the data by those third parties was scrutinised by the York Neuroimaging Centre's Research Governance Committee. The corresponding author may be contacted to initiate data sharing and assistance with seeking ethical approval for the use of the data.
